# Comparative evaluation of wastewater-treatment microbial fuel cells in terms of organics removal, waste-sludge production, and electricity generation

**DOI:** 10.1186/s40643-017-0163-7

**Published:** 2017-07-06

**Authors:** Yusuke Asai, Morio Miyahara, Atsushi Kouzuma, Kazuya Watanabe

**Affiliations:** 1 School of Life Science, Tokyo University of Pharmacy and Life Sciences, Tokyo, 192-0392 Japan; 2 Meidensha Corporation, Shinagawa, Tokyo, 141-8616 Japan

**Keywords:** Wastewater treatment, Microbial fuel cells, Activated sludge, Exoelectrogens, Power generation, Waste sludge

## Abstract

Microbial fuel cells (MFCs) are devices that exploit living microbes for electricity generation coupled to organics degradation. MFCs are expected to be applied to energy-saving wastewater treatment (WWT) as alternatives to activated-sludge reactors (ASRs). Although extensive laboratory studies have been performed to develop technologies for WWT-MFCs, limited information is available for comparative evaluation of MFCs and ASRs in terms of organics removal and waste-sludge production. In the present study, laboratory WWT experiments were performed using cassette-electrode MFCs and ASRs that were continuously supplied either with artificial domestic wastewater (ADW) containing starch and peptone or with artificial industrial wastewater (AIW) containing methanol as the major organic matter. We found that these two types of WWT reactors achieved similar organics-removal efficiencies, namely, over 93% based on chemical oxygen demands for the ADW treatment and over 97% for the AIW treatment. Sludge was routinely removed from these reactors and quantified, showing that amounts of waste sludge produced in MFCs were approximately one-third or less compared to those in ASRs. During WWT, MFCs continuously generated electricity with Coulombic efficiencies of 20% or more. In reference to ASRs, MFCs are demonstrated to be attractive WWT facilities in terms of stable organics removal and low waste-sludge production. Along with the unnecessity of electric power for aeration and the generation of power during WWT, the results obtained in the present study suggest that MFCs enable substantial energy saving during WWT.

## Background

Activated-sludge reactors (ASRs) are widely used for the treatment of domestic and industrial wastewater (Eckenfelder and O’Conner 1961). Although ASRs have been successfully used for wastewater treatment (WWT), intrinsic limitations associated with the use of ASRs include the consumption of large amounts of electric energy (Rosso et al. 2008) and the production of substantial amounts of waste sludge (Hall 1995). In Japan, annual electric power consumption in municipal WWT plants exceeds 80 billion KWh that accounts for approximately 0.7% of the total electric power consumption in this country (Mizuta and Shimada 2010). In addition, waste sludge annually produced in municipal WWT plants in Japan exceeds 70 million tons that accounts for over 20% of the total industrial waste (Imai et al. 2010).

Microbial fuel cells (MFCs) are devices that exploit living microbes for the conversion of organic matter into electricity (Logan et al. 2006). Using naturally occurring microbiomes, MFCs are able to generate electricity from organic wastes and wastewater (Watanabe 2008). In particular, MFCs are expected to be applied to energy-saving WWT (Li et al. 2014), and extensive work has been performed to develop MFC technologies applicable to WWT (Miyahara et al. 2013; Zhang et al. 2013). It has been demonstrated that ASRs can be converted to MFCs by removing aeration apparatuses from aeration tanks and inserting cassette-type electrodes instead (Yoshizawa et al. 2014). Merits expected in using MFCs for WWT include no need of energy for aeration, power generation from pollutants, and possible reduction in waste-sludge production. Although previous studies have evaluated organics-removal efficiencies and power generation in WWT-MFCs (Miyahara et al. 2013; Yoshizawa et al. 2014), limited experimental data are available for the amounts of waste sludge produced in MFCs (Zhang et al. 2013).

To date, there have been few studies that comparatively evaluated WWT performances of MFCs and ASRs operated under same conditions; in particular, it has not been experimentally demonstrated whether or not waste sludge produced in MFCs is actually less than that in ASRs. In the present study, we operated MFCs and ASRs in parallel by continuously supplying either with artificial domestic wastewater (ADW) containing starch, yeast extract, peptone, and urea as the major organic components or with artificial industrial wastewater (AIW) containing methanol as the major organic component, and their WWT performances (organics removal and waste-sludge production) were compared. Methanol was selected as a substrate in AIW, since it is widely used in industrial processes and known to be a major pollutant in industrial wastewater (Yamamuro et al. 2014). Results obtained are considered to serve as fundamental datasets for the practical development of MFC technologies for WWT.

## Methods

### Reactors used in WWT experiments

**Fig. 1:**
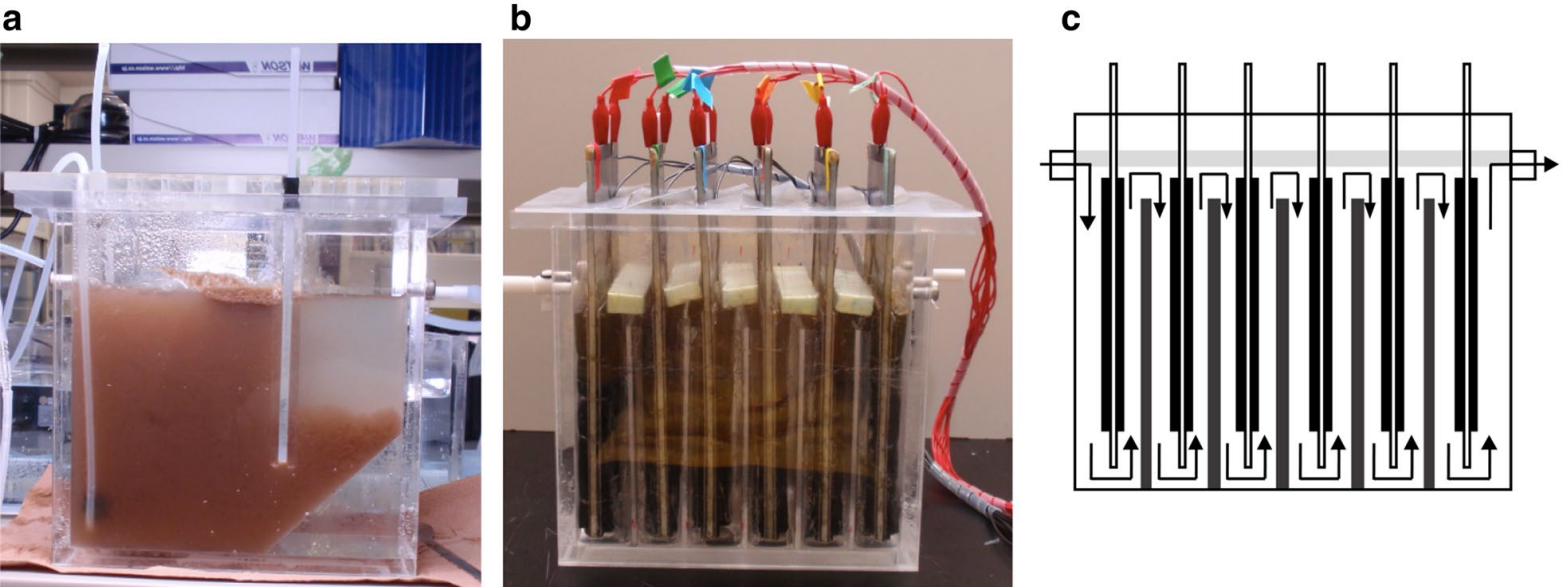
Reactors used in the WWT experiments. **a** A side view of ASR. **b** A side view of CE-MFC. **c** A schematic diagram of CE-MFC. Water flows are indicated with *arrows*. *Light gray bars* above the water surface are floating boards that prevent water from the contamination with oxygen, while *dark gray bars* between CEs are partition boards that facilitate the up and down flows of water

Photos of laboratory ASR and MFC used in the present study are presented in Fig. 1. An ASR comprised an aeration tank (approximately 1.5 L) and settling tank (approximately 0.5 L) that were separated by a partition board (Fig. 1a). Bottom parts of the aeration and settling tanks were connected, and sludge settled in the setting tank was returned to the aeration tank by gravity.

MFC used in the present study was a cassette-electrode (CE) reactor (approximately 1.5 L in water content; panels b and c in Fig. 1) that were equipped with 6 CEs prepared as described elsewhere (Shimoyama et al. 2008; Miyahara et al. 2013). As indicated with arrows in Fig. 1c, water flowed up and down between CEs and partition boards in CE-MFC. A CE had two anode/separator/cathode sets on both sides, between which air was filled (5 mm in thickness). An anode (126 cm^2^ in area) was made of a graphite-felt sheet (3 mm in thickness; Sohgoh Carbon, Yokohama, Japan), while a cathode (126 cm^2^) was an air cathode produced as described previously (Cheng et al. 2006). A separator (punctured polypropylene sheets, 1 mm in thickness) was used to separate between an anode and cathode. The water surface in CE-MFC was covered with floating boards to reduce the exposure to oxygen (Miyahara et al. 2015).

### Operation of WWT reactors

Sludge used as inocula for ASR and MFC was obtained from a return-sludge line in a municipal WWT plant (Asakawa Water Reclamation Center in Tokyo, Japan). Sludge was diluted with a mineral medium containing (per liter) 50 mg BBL yeast extract, 175 mg NH_4_Cl, 5.26 mg KH_2_PO_4_, 22.05 mg CaCl_2_·2H_2_O, 0.43 mg MgSO_4_·7H_2_O, 21.3 mg KCl, 8.76 mg NaHCO_3_, and 1 mL of trace-element solution (DSMZ 663; Deutsche Sammlung von Mikroorganismen und Zellkulturen GmbH) (pH 7.0), and the reactors were filled with the sludge suspension. An initial mixed-liquor suspended solid (MLSS) concentration was approximately 2000 mg L^−1^. The reactors were continuously suppled with either ADW containing starch, yeast extract, peptone, and urea as the major organic components (Miyahara et al. 2013) or AIW containing methanol as the major organic matter in the mineral medium. Chemical oxygen demand (COD, mg L^−1^) concentrations in ADW and AIW were approximately 500 and 1500 mg L^−1^, respectively. ADW with a COD value of 500 mg L^−1^ was used for comparing results obtained in the present study with those of previous studies (Miyahara et al. 2013; Yoshizawa et al. 2014). A hydraulic retention time (HRT, D) was 24 h in all experiments. ASR was supplied with air at a rate of 3 L min^−1^.

When commencing the operation of MFC, all anodes and cathodes were connected in parallel via an external resister (*R*_ext_, Ω), and a voltage across the resister (*E*, mV) was monitored using a data logger (HA-1510, Graphtec, Yokohama, Japan).

### Evaluation of organics removal

Effluents from the reactors were sampled at the effluent ports, and sludge was removed by centrifugation at 8000×*g* for 5 min. A COD concentration (mg L^−1^) in an effluent was measured using a COD reactor and a COD 0–1500 ppm range kit (Hach, Loveland, CO, USA). A COD-removal efficiency (CRE, %) was calculated from the influent COD (COD_in_, mg L^−1^) and effluent COD (COD_ef_, mg L^−1^) as ${\text{CRE}}\;{ = }\;\left[ {{\text{COD}}_{\text{in}} - {\text{COD}}_{\text{ef}} } \right]/{\text{COD}}_{\text{in}}$. Methanol in effluents was measured using gas chromatography (Yamamuro et al. 2014).

### Evaluation of waste-sludge production

MLSS in ASR was measured at certain intervals (generally 1 week). Before MLSS measurements, the partition board was removed, and sludge in ASR was uniformly suspended. A portion of the sludge suspension was sampled in triplicate, and, after being dried at 105 °C for 24 h, they were weighed. MLSS (mg L^−1^) was determined from the dry weights and a sample volume. In order to keep MLSS at 2000 mg L^−1^, an appropriate amount of sludge suspension was removed (removed sludge suspension; RSS, L) from ASR, and the fresh mineral medium was infused into ASR for compensating for the volume loss. Thereafter, the partition board was inserted again, and the operation of ASR was continued. During the operation, MLSS in effluent (MLSS_eff_; sludge was precipitated by the centrifugation and dried) was also measured by weighing dried suspended solids. Daily amounts of waste-sludge production (WSR, mg L^−1^ day^−1^) were calculated from MLSS and MLSS_eff_ as follows: WSR = {(MLSS × RSS)/Interval of measurement (*D*) + MLSS_eff_ × HRT}/Reactor volume.

To measure MLSS in MFC, the operation was temporarily halted at certain intervals. CEs were removed from the MFC reservoir, and loosely attached biofilms onto CEs were washed away by water flush and were mixed with the sludge suspension in the MFC reservoir. A portion of the mixed sludge suspension was sampled in triplicate, and, after being dried at 105 °C for 24 h, they were weighed. MLSS was calculated from the dry weight and a sample volume. The mixed sludge suspension was subsequently discarded, and, after MFC was equipped with the spent CEs and filled with the fresh mineral medium, the operation was re-started by supplying with ADW or AIW. During the operation, sludge in an effluent (precipitated by the centrifugation) was also measured at certain intervals as described above. Daily amounts of waste-sludge production were calculated from MLSS and MLSS_eff_ as described above.

### Evaluation of electricity generation in MFCs

Current (*I*, mA) was calculated from *E* and *R*_ext_ using equations $I = E/R_{\text{ext}} .$ A current density (*J*, mA m^−2^) was estimated by dividing *I* by the total projected area of the anodes (1512 cm^2^). Power (*P*, mW) was estimated according to an equation $P = IE,$ while a power density (PD, mW m^−2^) was estimated by dividing *P* by the total projected area of the anodes. A Coulombic efficiency (*ε*_c_, %) was calculated based on a COD removal (COD_in_ − COD_ef_) and a measured current as described previously (Miyahara et al. 2013). Polarization and power density curves were drawn using a potentiostat (HZ-5000, Hokuto Denko, Tokyo, Japan) as described previously (Miyahara et al. 2013), and the maximum power density (the peak in a power curve; *P*_max_, mW m^−2^; based on the projected anode area) and open-circuit voltage (OCV, mV) were determined as described elsewhere (Logan et al. 2006).

## Results

### COD removal

**Fig. 2:**
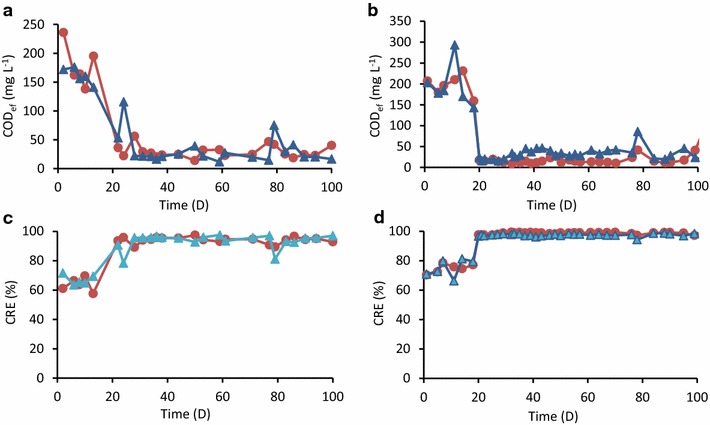
COD_ef_ (**a**, **b**) and CRE (**c**, **d**) during the operation of ASRs (*blue*) and MFCs (*orange*) treating ADW (**a**, **c**) and AIW (**b**, **d**)

ASR and MFC were continuously supplied with either ADW or AIW, and COD_ef_ was routinely measured (panels a and b in Fig. 2). During the operation of MFC, *R*_ext_ was changed as described below. Figure 2 shows that ~20 days were needed for ASR and MFC to sufficiently treat ADW and AIW, and the initial 20 days were therefore considered to be the acclimatization periods. These acclimatization periods were relatively long with unknown reasons. After the initial acclimatization periods, COD_ef_ values for ADW-treating ASR and MFC were mostly below 50 mg L^−1^ (Fig. 2a), and those for AIW-treating reactors were also below 50 mg L^−1^ (Fig. 2b). During these periods, methanol was not detected in effluents from the AIW-treating reactors (data not shown). Accordingly, CRE values for the ADW treatment were mostly over 90%, while those for AIW treatment was over 95%. During the stable operation (from days 80 to 100), mean CRE values were estimated (Table 1), showing that CRE values for ASR and MFC were not significantly different from each other.

**Table 1:** Summary of performance data obtained during stable operation (days 80 to 100)

Reactor	Wastewater	CRE (%)	Waste sludge (mg L^−1^ D^−1^)	*ε*_c_ (%)	*P*_max_ (mW m^−2^)	OCV (mV)
ASR	ADW	94 ± 5	44 ± 15	–	–	
	AIW	97 ± 4	88 ± 14	–	–	
MFC	ADW	93 ± 4	10 ± 2	26 ± 5	124 ± 11	770 ± 23
	AIW	98 ± 2	30 ± 4	20 ± 3	160 ± 10	780 ± 26

Values are means ± SDs (*n* > 3)

### Waste-sludge production

**Fig. 3:**
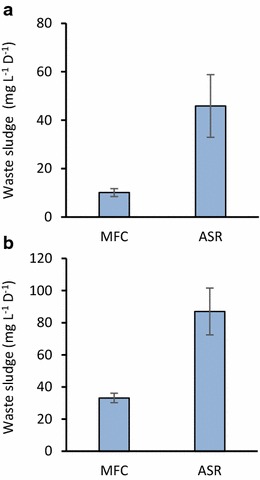
Daily waste-sludge production in ASRs and MFCs treating ADW (**a**) and AIW (**b**). *Bars* represent means (*n* = 3), while *error bars* are SDs

At certain intervals, sludge suspensions were removed from ASR and MFC as described in the “Methods” section, and amounts of waste sludge removed from these reactors (waste sludge) were quantified. Figure 3 shows normalized amounts of waste sludge produced during the stable operation of ASR and MFC (from day 80 to day 100) treating either ADW or AIW. It was found that the amounts of waste sludge produced in MFC were significantly lower than those in ASR. The amount of waste sludge produced in ADW-treating MFC (ADW-MFC) was approximately one-third of that in ADW-treating ASR, while that in AIW-treating MFC (AIW-MFC) was approximately one-fifth of that in AIW-treating ASR (Table 1).

### Electricity generation in MFC

**Fig. 4:**
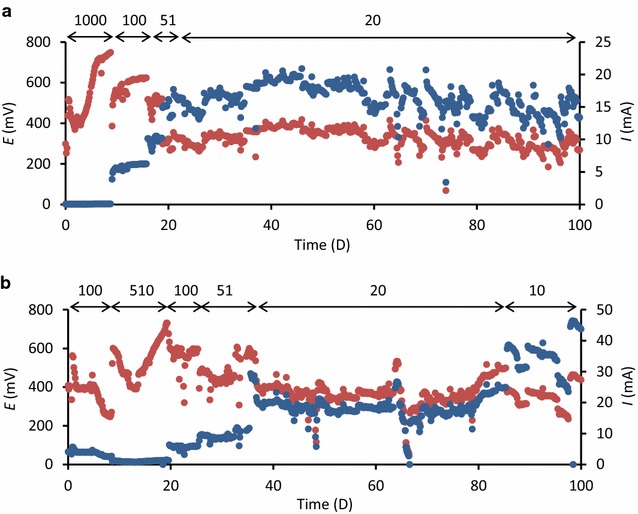
*E* (*orange*) and *I* (*blue*) during the operation of ADW-MFC (**a**) and AIW-MFC (**b**). *Arrows* and *numbers* above the graphs indicate *R*_ext_ for these MFCs

Changes in *E* for ADW-MFC and AIW-MFC were monitored using data loggers (Fig. 4), and *I* was calculated from *E* and *R*_ext_ (Fig. 4). As presented in this figure, *R*_ext_ was changed to maintain *E* at around 400 mV; previous studies have shown that efficient WWT and electricity generation were achieved by maintaining *E* at around such values (Miyahara et al. 2013; Yoshizawa et al. 2014). As a result, *R*_ext_ for ADW-MFC was finally kept at 20 Ω, while that for AIW-MFC was 10 Ω. This figure shows that AIW-MFC generated more current than ADW-MFC in the last 20 days. Based on measured *I* values and COD removal, *ε*_c_ was determined as an index for electron recovery (Table 1). The mean *ε*_c_ value for ADW-MFC was higher than that for AIW-MFC, despite that more current was generated in AIW-MFC than that in ADW-MFC. This was due to the high COD load to AIW-MFC compared to ADW-MFC.

**Fig. 5:**
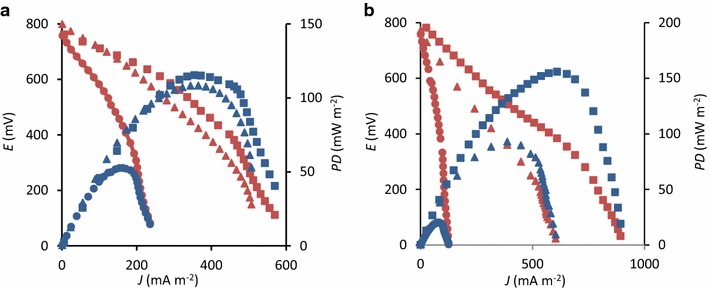
Representative polarization (*orange*) and power curves (*blue*) for ADW-MFC (**a**) and AIW-MFC (**b**). *Circles* day 20, *triangles* day 60, *squares* day 100

**Fig. 6:**
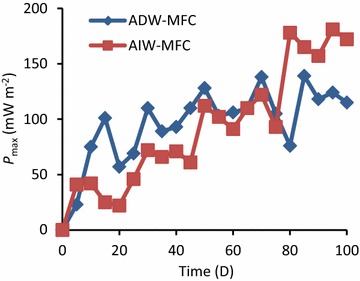
Changes in *P*_max_ during the operation of ADW-MFC and AIW-MFC

Polarization analyses were routinely conducted (generally once in every 5 days) for monitoring changes in electrochemical performances of ADW-MFC and AIW-MFC. Representative polarization and power curves are presented in Fig. 5, and changes in *P*_max_ for these MFCs are illustrated in Fig. 6. Figure 5 shows that ADW-MFC and AIW-MFC were properly functioned as fuel cells that produced catalytic currents. We therefore employed *P*_max_ as an index for monitoring electrochemical performances of these MFCs. As indicated in Fig. 6, *P*_max_ relatively rapidly increased in ADW-MFC compared to that in AIW-MFC, whereas higher *P*_max_ values were finally observed for AIW-MFC than those for ADW-MFC. This trend would have been related to the minor occurrence of anaerobic methanol-dissimilatory microbial populations in the activated sludge used as the inoculum for MFCs. The competition between denitrifiers and exoelectrogens also needs to be considered. It is also likely that the final high power density observed for AIW-MFC was due to the high COD in AIW compared to that in ADW. A possible reason for inconsistency in trends between the COD removal and *P*_max_ would be that microbial terminal electron-accepting reactions other than current generation also contributed to the COD removal, as indicated with *ε*_c_.

## Discussion

ADW used in the present study was the same as that used in our previous laboratory experiments for assessing CE-MFCs for WWT (Miyahara et al. 2013; Yoshizawa et al. 2014); in these studies, however, despite that HRT and COD_in_ were also the same as those in the present study, CRE values were reported to be around 80%. On the other hand, as presented in Fig. 1 and Table 1, CRE values observed in the present work were higher than 90% (93% in average). We consider that the up and down flow of water in MFC was effective for improving the quality of effluent water; this flow system was newly employed in the present study. We had employed right and left flows of water in CE-MFC (named the slalom flow) in previous studies (Miyahara et al. 2013; Yoshizawa et al. 2014), while we found that this system was associated with a problem of undercurrent that was not effectively contacted with electrode surfaces. The present study therefore proposes that the up and down flow of wastewater is effective for gaining high WWT efficiencies in CE-MFCs. In order to further improve water flow in CE-MFCs, computational fluid dynamics analyses may be necessary.

It has long been predicted that waste sludge produced in MFC during WWT may be much less than that in ASR (Oh et al. 2010; Li et al. 2014), while limited information is available for amounts of sludge produced in MFCs (Zhang et al. 2013). Zhang et al. (2013) monitored suspended solids in MFCs treating municipal wastewater, while amounts of waste sludge produced in MFC were not precisely compared with that in ASR operated under same conditions. To our knowledge, the present study was the first to empirically evaluate waste-sludge production in MFC and ASR operated under same WWT conditions, demonstrating that substantial reduction in waste-sludge production is possible in MFC compared to that in ASR.

Previous studies have demonstrated that excess biofilms, particularly those loosely adhere to reservoir and electrode surfaces, should be removed for maintaining the electricity-generation capacity of continuous-flow MFCs (Miyahara et al. 2013). These biofilms are the major components in waste sludge in MFCs, and it is therefore important to understand how much biofilm is produced in MFCs during WWT, and also how often these biofilms should be removed from MFC inner surfaces. Despite that the present study routinely removed biofilms and discarded them as waste sludge, electricity generation was kept relatively constant in ADW-MFC and AIW-MFC. These results support the idea that excess biofilms (mostly not involved in electricity generation) should be routinely removed for maintaining electric outputs from WWT-MFCs, and the present study provides with a benchmark for sludge-removal practices for WWT-MFCs. Further studies will be necessary to develop protocols to determine an appropriate frequency of sludge removal from WWT-MFCs depending on wastewater qualities.

Concerning electric outputs from MFCs, values for *ε*_c_ and *P*_max_ reported in the present study were similar to those reported in our previous studies despite the different water-flow systems (Miyahara et al. 2013; Yoshizawa et al. 2014). Although the mean *ε*_c_ values of 20 and 26% reported in the present study (Table 1) are relatively high compared to those for other WWT-MFC experiments, e.g., approximately 10% for MFC treating municipal wastewater (Zhang et al. 2013), further increases in *ε*_c_ are desirable for developing more useful MFCs. Since a previous study has shown that large portions of electrons released from organics by exoelectrogens are consumed by aerobic respiration using oxygen that entered through air cathodes in MFCs (Shimoyama et al. 2008), improvement of air cathodes (e.g., optimization of the oxygen-transfer rate) will be necessary for further improving *ε*_c_ in WWT-MFCs.

## Conclusions

The present study comparatively evaluated MFCs and ASRs using two different types of wastewater, suggesting that MFCs are attractive WWT facilities in terms of stable organics removal and low waste-sludge production. Along with the unnecessity of electric power for aeration and the generation of power during WWT, the results obtained in the present study further suggest that MFCs are able to save substantial amounts of energy needed for WWT. Although the possibility for energy saving in wastewater-treatment MFCs has already been suggested (Li et al. 2014), the results presented in this study (in particular, those related to waste-sludge reduction), for the first time, facilitate reasonable estimation of energy saving in wastewater-treatment MFCs. In municipal WWT plants, electric energy is typically used for aeration (approx. 40%), sludge treatment (approx. 30%), and others (30%) (Shi 2011). On the other hand, electric energy generated in MFC during the treatment of typical municipal wastewater (e.g., COD 200 mg L^−1^) is estimated to be equivalent to 20% of the energy needed for WWT in municipal plants. It is therefore suggested that the use of MFC for WWT can save approximately 80% in total (a sum of 40% from no aeration, 20% from 2/3 sludge reduction, and 20% from electricity generation) of energy consumed in ASR-type WWT plants. For practical application of MFCs to WWT, further studies are necessary for developing technologies for the production of large and cheap electrodes. Experiments using real wastewater and those with short HRT should also be done.
